# Reviewers in 2017

**DOI:** 10.1098/rsos.180228

**Published:** 2018-03-07

**Authors:** Jeremy K. M. Sanders

**Affiliations:** Editor-in-Chief

Last year, *Royal Society Open Science* published its first list of all reviewers (other than those who opted out) who had volunteered their time and expertise to referee manuscripts submitted to the journal [1]. As a form of public recognition of their service, the list proved very popular with our reviewers.

With this in mind, we have decided to make publication of the names of our reviewers an annual event. Below, we publish the names (and ORCID where it has been provided) of all our reviewers who wished to receive this public recognition.

On behalf of all the Editors and editorial office, I thank all of our reviewers past, present and future, and we look forward to your continued support of *Royal Society Open Science*.

Aamer, A
Aarts, G
Aassime, A
Abanda, H
Abdel-Aal, H
Abecassis, B
Abourachid, A https://orcid.org/0000-0002-7238-2795
Acien, G
Adamatzky, A
Adamczyk, P
Adami, C
Aerts, P http://orcid.org/0000-0002-6867-5421
Agnolin, F http://orcid.org/0000-0001-5073-561X
Agui, T
Aguilar-Perera, A
Aguirre, JD
Aharonov, Y
Ahmadi, A
Ahmadi, MH
Ahn, A
Ahnesjö, I
Aho, K
Ahyong, ST
Aiello, L
Airoldi, C
Akbarzadeh, A
Akcay, E http://orcid.org/0000-0001-8149-7124
Akhavan, O
Akhtar, I
Akins, C
Akkutlu, IY
Alavi, A
Alavi, S
Albani, J-R
Albarella, U
Alberic, M
Albert, J
Albiach Serrano, A
Albino, A
Alda, J
Allen, D
Allgower, F
Allison, A
Altmann, E http://orcid.org/0000-0002-1932-3710
Altshuler, D
Alvarez-Buylla, E
Amack, J http://orcid.org/0000-0002-5465-9754
Amaral, L
Amato, K
Ambike, S
Ameel, B
Ameziane, N
Amigo, J http://orcid.org/0000-0002-1642-1171
An, Y
Anadon, J
Anandhakumar, S
Andrada, E http://orcid.org/0000-0002-9300-4056
Andrade, M http://orcid.org/0000-0002-2931-5378
Angelsky, O
Anné, J http://orcid.org/0000-0002-6838-4317
Ansaloni, S
Anttila, J
Aoi, S
Aoki, K http://orcid.org/0000-0002-8426-4091
Appleby-Thomas, G http://orcid.org/0000-0002-9129-1995
Apps, C
Apthorp, D
Araujo, M
Araujo, NAM
Arbilly, M http://orcid.org/0000-0002-3227-882X
Arbour, V
Arbuckle, B
Arcaute, E
Arciuli, J http://orcid.org/0000-0002-7467-9939
Arlettaz, R
Armas, O
Armbruster, J
Artigas, P
Arulsamy, AD http://orcid.org/0000-0002-1088-2559
Aryal, A http://orcid.org/0000-0002-2695-9901
Asahara, M
Asami, T
Asbury, K
Asgharipour, MR
Ashby, B
Aslan, A
Astumian, D
Astumian, R
Atar, FB
Atkinson, D
Attasart, P
Aubret, F
Audoly, B
Ault, A
Ausloos, M http://orcid.org/0000-0001-9973-0019
Avarguès-Weber, A
Avenanti, A
Avin, S
Avramopoulos, A
Ayyash, M
Azabou, S
Azad, AK
Azmi, A
Azodi Avval, R
Bachmann, T
Bachor, H
Backstrom, N
Baer, C http://orcid.org/0000-0002-0140-5814
Baeza-Romero, M
Bag, S
Bagrow, J
Bainbridge, W
Baird, R
Baka, M
Baker, C
Baker, D http://orcid.org/0000-0002-0161-443X
Baker, J
Balasubramaniam, R
Balch, AL
Balci, F
Baldacchino, F
Balderston, N
Balebona, M
Balietti, S
Ballini, R
Balshine, S
Baltzinger, C
Baltzopoulos, B
Bandara, N
Banerjee, J
Banerjee, P
Baños, R http://orcid.org/0000-0001-7844-4902
Bañuelos, J
Barbosa, M http://orcid.org/0000-0003-0327-9580
Barboutis, C
Bard, K
Bardi, U
Barker, D
Barnes, F
Barnes, J
Barnes, M http://orcid.org/0000-0002-5550-8587
Baron, A
Barons, M https://orcid.org/0000-0003-1483-2943
Bar-Oz, G
Barraquand, F
Barreiro, A http://orcid.org/0000-0003-0286-4139
Barreto, M
Barrientos, R
Barsky, D
Barton, M
Basak, S
Bastakoti, B
Bateson, M http://orcid.org/0000-0002-0861-0191
Batiste, O
Battiston, F http://orcid.org/0000-0001-9646-6232
Batty, M
Bauer, R
Baumann, P
Bazar, G
Beaty, L
Becerro, A
Becher, P
Beebe, B
Beekman, A
Beierholm, U
Beisner, B
Belchior, J
Bell, H
Bellstedt, D
Beltran, A
Ben-Arieh, D
Benedict, C
Benjamin, C http://orcid.org/0000-0001-9794-243X
Bennie, J
Bensch, S
Benson, J http://orcid.org/0000-0002-3993-4340
Benson, R
Bentkowski, P http://orcid.org/0000-0002-1220-1075
Bentley, W
Bergaentzle, M
Berggren, P
Bergman, R
Bergman, T
Bernard, K
Berns, G
Berry, D
Bert, T
Beshers, S
Besier, T
Besley, N http://orcid.org/0000-0003-1011-6675
Best, J
Bestmann, S
Bever, G http://orcid.org/0000-0002-8590-584X
Bex, P
Bhaduri, G
Bhattacharjee, CR
Bianchi, A
Bieler, R
Bigan, E
Bigot-Clivot, A
Bijak, J
Bikas, R
Biler, P
Billard, L
Birn-Jeffery, A
Bishop, J
Bizzarro, J
Blagrove, M
Blamires, S http://orcid.org/0000-0001-5953-3723
Blanco, E
Block, P
Blomberg, C
Blyuss, K
Bobbert, M
Boehm, C
Boehmer, C
Boek, E
Boessenecker, R http://orcid.org/0000-0002-6117-128X
Bolsen, T
Bolsterlee, B
Bona, P http://orcid.org/0000-0001-7782-855X
Bonham-Carter, O
Bonnet, F http://orcid.org/0000-0003-2268-5947
Bootsma, M
Borge-Holthoefer, J
Borregaard, M
Bose, N
Bosi, E http://orcid.org/0000-0002-4769-8667
Botta, F http://orcid.org/0000-0002-5681-4535
Boucherie, P
Bowern, C
Bowers, D
Bowers, E http://orcid.org/0000-0003-4488-1463
Boyer, D
Boyer, D
Boyle, A
Bradbury, I
Branch, T
Brandt, M
Brar, S
Braze, D
Bredeche, N http://orcid.org/0000-0002-8241-7461
Brennan, P
Brennen, G
Bretti, G
Brewer, M
Brewer, R
Briffa, M http://orcid.org/0000-0003-2520-0538
Brigham, R
Brinkløv, S
Briscoe, J
Brochu, C
Broersen, K
Brooks, M
Broom, M
Brothers, C http://orcid.org/0000-0001-6942-0925
Brown, C
Brown, C
Brown, J
Brown, S
Brownbill, P
Browne, D
Brown-Schmidt, S
Brucato, N
Bruskotter, J
Brust, V
Brysbaert, M
Budhi, S
Buehlmann, C
Bugnyar, T
Bujorianu, M
Bulgarella, M
Bulgariu, L
Burdick, M
Burgert, I
Burrage, K
Burton, C
Burton, N
Buzatto, B
Cacucci, F
Cada, G
Cade, D
Cadena, E
Caillaud, D
Caldarelli, G
Calmet, X
Camacho, JP
Cameron, E
Cameron, J
Cameron, S
Camp, J
Campagnolo, P http://orcid.org/0000-0002-8957-3969
Campbell, O
Campos, D http://orcid.org/0000-0003-1982-3288
Canals, M
Capdevila-Cortada, M
Capel, C
Capraro, V http://orcid.org/0000-0002-0579-0166
Capriles, J
Cardé, R
Cárdenas, J
Carey, D
Carleton, K
Carré, R http://orcid.org/0000-0002-9219-0686
Carrillo, J
Carter, A http://orcid.org/0000-0002-8359-2085
Casareto, B
Casaroli, G
Casas, J
Caseri, W
Casey, E
Cashman, K
Cassese, A http://orcid.org/0000-0001-5830-4136
Cassill, D http://orcid.org/0000-0002-5234-6228
Cassini, G
Casson, A
Castellani, B
Castellano, S
Castellote, M
Castillo Castillo, A
Castle, GSP
Castles, M
Cava, C
Cazzola, D
Ceccarelli, S
Ceccatto, V
Ceia, F
Ceylan, H
Cezilly, F https://orcid.org/0000-0003-2673-6713
Cha, S
Chadwick, N
Chahl, J
Chakrabarti, B http://orcid.org/0000-0002-6649-7895
Chan, D
Chan, KYK http://orcid.org/0000-0002-7775-4383
Chan, T-Y
Chandrashekar, CM
Chang-Jian, C-W
Chao, J
Chapeau-Blondeau, F
Chapman, N
Chapple, T
Chatterjee, A
Checa, A http://orcid.org/0000-0001-7873-7545
Chemat, F
Chen, F
Chen, G
Chen, G
Chen, J
Chen, J
Chen, J http://orcid.org/0000-0002-1277-7891
Chen, M
Chen, N
Chen, R
Chen, S
Chen, S
Chen, T
Chen, W
Chen, X
Chen, X
Chen, X http://orcid.org/0000-0001-5027-4147
Chen, X
Chen, X
Chen, Z
Cheng, K
Cheng, M
Chiang, W-C (Riyar)
Chin, D
Chmely, S
Choe, Y
Chopin, J
Chouinard-Thuly, L http://orcid.org/0000-0002-9131-1191
Choukchou-Braham, A
Christensen-Dalsgaard, J
Christianini, A
Chrobok, A
Chu, C-J
Church, R
Chutipong, W
Cicchini, GM
Cifelli, R
Cihan, AF
Cimino, M
Cingi, H
Clairambault, J
Clapham, P http://orcid.org/0000-0002-2776-5746
Claramunt, R
Clark, A http://orcid.org/0000-0001-5908-2862
Clark, C http://orcid.org/0000-0002-7692-8150
Clark, J
Clark, L
Clarke, A
Clarke, R
Clausen, L
Clavien, C
Clay, Z http://orcid.org/0000-0002-3016-1732
Clayton, C
Clegg, J
Cleland, T http://orcid.org/0000-0001-9198-2828
Cleuren, B
Clifford, C
Clout, M
Clua, E
Codd, J http://orcid.org/0000-0003-0211-1786
Coggan, J
Cohen, A
Coimbatore Balram, K
Cole, J
Cole, M
Coleman, R
Collard, M
Collett, T
Colman, M
Colombi, C
Colquhoun, D http://orcid.org/0000-0002-4263-017X
Coltheart, M
Comer, P
Connahs, H
Conradi, C
Constable, G
Conway, J
Cook, A
Cooper, C
Cooper, J
Cooper, R
Corkeron, P
Corlett, P
Cornuault, P-H
Corominas-Murtra, B
Correia, I
Corso, G
Corthals, A
Costas Rodriguez, M
Costidis, A
Costine, A
Cotel, A
Cracraft, J
Craig, DQM
Crandall, K
Crane, A http://orcid.org/0000-0002-8645-8180
Crasto, C
Crauste, F
Crawford, R
Creel, S
Cremer, S
Crespi, B http://orcid.org/0000-0002-0362-392X
Crisponi, G http://orcid.org/0000-0002-3902-0231
Cristol, D
Croatt, M
Crofts, S
Cronin, A
Cronk, L
Crooks, K
Crowley, J http://orcid.org/0000-0002-3364-2267
Cui, X
Cui, Y
Cullen, P
Cully, A
Cuminato, JA
Cummings, M http://orcid.org/0000-0002-4873-5378
Cummins, F
Cunill, F
Cunningham, SJ
Cupello, C http://orcid.org/0000-0001-6030-8670
Currie, A
Currie, C
Currie, T
Curtis, V
Cushman, F
Cypher, B
Czaczkes, T http://orcid.org/0000-0002-1350-4975
D'Orsogna, MR http://orcid.org/0000-0003-2828-9523
da Paixao Seva, A
Dag, S
Dahanukar, N
Dahl, S
Dalcanale, E
Dalerum, F
Dalvi, S
Damian, D
Dangerfield, C
Daniels, S
Danielsbacka, M
Danovaro, R
Darling, J
Das, P
Das, S
Dasgupta, S
Dassanayake, R
Davies-Thompson, J
Davis, K
Davis, R
Dawkins, L
Day, I
de Aguiar, M
De Aza, PN
de Bekker, C http://orcid.org/0000-0002-7325-172X
De Benedictis, P
de Boer, B
De Jong, D
De Kimpe, N
de Lazaro, S
De Martinis, V
De Paepe, W
De Sy, V
Dean, M http://orcid.org/0000-0002-5026-6216
Dearnley, P
Dediu, D
Deeming, DC http://orcid.org/0000-0002-9587-6149
Del Vicario, M
Delahaie, B http://orcid.org/0000-0002-4250-2266
Delamater, A
Delgado, M http://orcid.org/0000-0002-3252-2618
Dell'Olio, F
Dellwo, V
Delmelle, E
DeMiguel, D
Demkowicz, S
Demos, T
Deng, H
Dennis, T
Dentz, M
Depuydt, D
Dermauw, W http://orcid.org/0000-0003-4612-8969
Deska, J
DeVita, P
Dexter, K
Dezecache, G http://orcid.org/0000-0002-9366-6287
Dhaliwal, GS
Dhami, N
Di Talia, S
Diaz, R
Dick, C
Dickson, B http://orcid.org/0000-0001-6299-5224
Dickson, B
Diekelmann, S
Dierssen, H
Diesendruck, C
Dietl, G http://orcid.org/0000-0003-1571-0868
Difford, M
Dimitratos, N
Ding, F
Ding, S
Ding, Y
Dinis, L
Diquelou, M
Distiller, G
Doblaré, M
Docampo, R
Dodson, P https://orcid.org/0000-0001-9059-1155
Doll, A
Domenici, P http://orcid.org/0000-0003-3182-2579
Dominguez, C
Dominici, S
Dominy, N http://orcid.org/0000-0001-5916-418X
Dong, C
Dong, W
Dong, Y
Dong, Y
Donnelly, C
Donner, R
Donovan, S
Doran, C
Dorfman, K http://orcid.org/0000-0003-0065-5157
Douglas, M http://orcid.org/0000-0001-9670-7825
Downs, R
Draganoiu, T
Drake, KK
Drasdo, D
Drengstig, T
Dreosti, E
Drumheller, S
Drury, J http://orcid.org/0000-0002-7748-5128
Du, D
Du, M
Du, M
Du, X
Duan, H
Duan, W
Duan, Y
Duanmu, Z
Duarte, J
Duboscq, J http://orcid.org/0000-0002-6342-6709
Dugatkin, L
Dugnoille, J
Dullemond, C
Duman, O
Dunbar, R http://orcid.org/0000-0002-9982-9702
Dunlop, R http://orcid.org/0000-0002-0427-6317
Dunn, S-J
Dunningham, J
Duraisamy, C
DuRant, S
Durrant, S http://orcid.org/0000-0003-3659-179X
Dushoff, J
Duthie, B
Duvall, C
Dzierzbicka, K
Eanes, W
Easdown, D
Ebensperger, L
Eckert, K
Edwards, G
Edwards, P
Efferson, C
Egger, B
Ehlers, J
Ehrhardt, M
Ekdale, E
Elias, D
Elias, M http://orcid.org/0000-0003-0066-6542
Ellis, R http://orcid.org/0000-0002-3117-0075
Ellis, S http://orcid.org/0000-0001-9019-6040
Elsey, R
Elwood, R
Embke, H
Enders, E
Enriquez, J
Eppley, T
Er, E
Eran, A
Ercan, N
Erickson, G
Eriksen, N
Eriksson, A
Eriksson, K
Ernest, H http://orcid.org/0000-0002-0205-8818
Escobedo Bonilla, C
Esposito, A
Essex, C
Esteves da Silva, JC
Esteves, P
Etchells, P
Evano, G
Evans, S
Everman, E http://orcid.org/0000-0002-3190-4171
Evin, A http://orcid.org/0000-0003-4515-1649
Ewing, L http://orcid.org/0000-0001-5263-1267
Eyestone, W
Fabricius, K http://orcid.org/0000-0001-7671-4358
Falcone, E http://orcid.org/0000-0002-9401-1354
Falko, V
Fan, H
Fan, X
Fan, Y
Fang, P
Fang, X
Fanny, C
Fantuzzi, N
Farine, D http://orcid.org/0000-0003-2208-7613
Farji-Brener, A
Farmer, R
Farrell, A
Farris, D http://orcid.org/0000-0002-6720-1961
Fast, M
Fathima, N
Faulkner, G
Favier, L
Fay, D
Feinman, G
Feldman, M http://orcid.org/0000-0002-0664-3803
Feller, K
Fenelon, J
Feng, H
Feng, H
Feng, Q
Feng, X
Fenton, A
Fenton, B
Ferguson, C
Fernandes Mendes, M
Fernández-Herrera, M
Fernández-Lafuente, R
Ferrando, R
Ferrante, E
Fessler, D
Feuerbacher, E
Fey, N
Fichtel, C
Field, K http://orcid.org/0000-0002-5196-2360
Field, R
Fielding, J
Figner, B
Filingeri, D http://orcid.org/0000-0001-5652-395X
Filippi, P http://orcid.org/0000-0002-2990-9344
Fink, J
Finlayson, N
Fiorelli, L
First, M
Firth, J http://orcid.org/0000-0001-7183-4115
Fischer, J
Fisher, A
Fisher, D
Fitch, T
Fitt, B
Fitzgerald, D
FitzPatrick, W http://orcid.org/0000-0002-6922-1181
Flasche, S
Flechsig, G-U
Fleischer, R
Fleming, C
Fleming, P
Fletcher, J
Floccia, C https://orcid.org/0000-0003-3931-9791
Flor, H
Floria, L
Fogarty, M
Fontaine, C
Fordyce, RE
Forkman, B
Forslund, K
Foster, D
Fouchet, D
Fournier, AMV http://orcid.org/0000-0002-8530-9968
Fowler, P
Fozard, J http://orcid.org/0000-0001-9181-8083
Franc, A
Franchi, N
Francis, G http://orcid.org/0000-0002-8634-794X
Franco-Villafañe, JA
Frank, M
Fransozo, V
Franz, J http://orcid.org/0000-0001-9523-9708
Fraser, D
Fraser, K
Frasier, C
Frederiksen, M
Freitak, D
Frick, P
Friedman, M
Fritz, L
Fröbisch, J
Fry, E
Fu, J
Fu, S http://orcid.org/0000-0001-5680-7493
Fu, Y
Führer, H-P
Fujita, K
Full, R
Funnell, N
Furla, P
Furuichi, T
Fushimi, RR
Gade, N
Gadipelli, S http://orcid.org/0000-0002-1362-6905
Gago, R
Gaillard, J-M
Galanti, N
Gale, A
Galipaud, M
Galla, T
Galvão, D
Gammaitoni, L
Gan, N
Gan, Z
Ganesh, A
Ganeshpandian, M
Gangestad, S
Ganghoffer, J-F
Ganswindt, A
Gante, H
Gao, H http://orcid.org/0000-0001-6852-9435
Gao, Y
Garcia, G
Garden, D
Gardner, A http://orcid.org/0000-0002-1304-3734
Gardner, J http://orcid.org/0000-0002-1430-4350
Garg, P
Garland, E
Garnham, A
Garrote, G
Gasser, C
Gassón, R
Gatta, D
Gavrilets, S
Gawron, P
Gazzano, A
Gear, W
Geffen, E http://orcid.org/0000-0002-3028-0045
Geiselhardt, S
Generalov, I
Gentili, R
Georges, A http://orcid.org/0000-0003-2428-0361
Georgieva, E
Gerdol, M
Gerelli, Y
Gerlee, P
Germain, D
Germain, PL
Gerson, A
Gerstein, A
Gerwick, L
Gerwing, T http://orcid.org/0000-0002-4433-1843
Gethings, O
Ghalambor, C
Ghayour-Mobarhan, M
Ghirlanda, S http://orcid.org/0000-0002-7270-9612
Ghobadi, TGU
Giarra, M
Gibbons, M
Gibson, E
Gilbert, P
Giles, M
Giles, S
Gillivray, B
Gilmore, K
Ginelli, F
Gingins, S
Giorgi, M
Giribet, G
Gleason, T
Godfrey, S
Godfrey, S
Godwin, J
Golbuu, Y
Goldstein, E
Gómez, C
Gomez, G
Gomez-Lievano, A http://orcid.org/0000-0001-8320-0857
Gonçalves, LMG
Gong, M
Gonzalez, A
Gooch, K
Goodacre, S
Goode, D
Goodwin, S
Goos, J
Göpfert, M
Gorban, A http://orcid.org/0000-0001-6224-1430
Gordo, O
Gorecki, J
Gorochowski, T
Gorokhova, E http://orcid.org/0000-0002-4192-6956
Goswami, A
Goswami, S
Gotz, T
Gould, V
Gowers, T
Grabowska, B
Grace, D http://orcid.org/0000-0002-0195-9489
Grady, J
Graham, J
Graham, J
Granger, P
Gräter, F
Graves, W
Gravish, N
Gray, K http://orcid.org/0000-0002-6071-4588
Gray, R
Grebenkov, D
Greco, L
Green, J
Green, S
Greenebaum, B
Greenhouse, I
Gregorič, M
Gregory, R http://orcid.org/0000-0001-7900-7501
Greig, A
Grelier, S
Grenning, A
Griebel, U
Grieneisen, L
Grier, D http://orcid.org/0000-0002-4382-5139
Griffin, A https://orcid.org/0000-0003-4624-9904
Griffin, L http://orcid.org/0000-0001-6286-2018
Griffiths, R
Grillo, F
Grima, R http://orcid.org/0000-0002-1266-8169
Grimes, D
Grimes, R
Grinfeld, M
Grismer, L
Grond, K
Gronenberg, W
Gronlund, S
Grorud-Colvert, K
Grosbras, M-H
Gross, J
Gross, T
Grossmann, I
Gu, H
Gu, J
Guan, X
Guan, YJ
Gude, V
Guerenstein, P
Guerrero Garcia, G
Gulcin, I
Guler, MO http://orcid.org/0000-0003-1168-202X
Gumm, J
Gumus, ZP
Gumuscu, B
Gunji, Y http://orcid.org/0000-0002-0462-4932
Güntürkün, O
Guo, X
Guo, C
Guo, H
Guo, K
Guo, K http://orcid.org/0000-0001-6765-1957
Guo, Q
Guo, Y
Guo, Z
Gupta, HS http://orcid.org/0000-0003-2201-8933
Gürsoy, U
Gustison, M
Gutub, A
Haddock, S
Hagen, S
Haiges, R
Halim, T
Hall, M http://orcid.org/0000-0002-1263-8314
Hall, N
Halliday, D
Hamby, K
Hamid, S
Hamilton, M
Han, M-Y
Han, YL
Hancock, E
Hanlon, R http://orcid.org/0000-0003-0004-5674
Hansson, B
Harasti, D http://orcid.org/0000-0002-2851-9838
Harbach, R http://orcid.org/0000-0003-1384-6972
Harper, J
Harris, K
Harrison, D
Harrison, H https://orcid.org/0000-0001-8831-0086
Hart, A
Hasanov, N
Hasell, T
Hasenjager, M http://orcid.org/0000-0003-2193-4120
Haslinger, S http://orcid.org/0000-0003-0790-1701
Hassan, W http://orcid.org/0000-0001-9703-4116
Hassanabadi, H
Hassel, K
Hast, M
Hatchwell, B
Hauser, D http://orcid.org/0000-0001-8236-7372
Hauss, T
Havlíček, J
Hayden, B
Hays, G http://orcid.org/0000-0002-3314-8189
He, K
He, L
He, N
He, X
Heathcote, R
Hechenleitner, M http://orcid.org/0000-0002-9538-5681
Hecnar, S
Hedrick, P
Heffernan, K
Heiligers, J
Heinze, S
Held, L
Helle, S http://orcid.org/0000-0002-6216-3759
Hellweger, F
Hemelrijk, C
Henderson, T http://orcid.org/0000-0002-5028-9047
Hendy, S
Henningsson, P
Hensel, R
Heritage, J http://orcid.org/0000-0002-8603-6447
Hernandez-Lemus, E
Herrel, A
Hertz, H
Hessen, D
Hetem, R
Hewitt, R
Hickerson, C-A
Hideko, K
Hill, A
Hill, C
Hill, R
Hills, P
Hills, R
Hino, H
Hippler, M
Hirst, J
Ho, J
Hodo, W
Hoelzel, R
Hoffmann, KH
Hohn, A
Holtz, T
Homans, F
Hone, D
Honey, M
Honey, R
Hoogesteijn, A
Hope, L
Hopkins, C
Hopkins, N
Hopkins, S
Hopkins, W
Hore, P http://orcid.org/0000-0002-8863-570X
Hori, Y http://orcid.org/0000-0002-3253-4985
Horn, A http://orcid.org/0000-0002-0695-6025
Hornsby, A
Horvat, J
Horvath, G http://orcid.org/0000-0002-9008-2411
Hosken, D http://orcid.org/0000-0003-2508-3909
Hossain, M
Hosseini Araghi, A
Hotchkin, C
Hou, WG
Hou, X-G
House, T http://orcid.org/0000-0001-5835-8062
Houslay, T
Howes, J
Howlett, B
Hoz, S
Hozumi, A
Hruschka, D
Hu, J
Hu, M
Hu, X
Hu, Y
Hu, Y
Huang, C
Huang, H
Huang, J
Huang, J
Huang, M
Huang, Y
Huang, Y
Huang, Y
Huang, Y
Huang, Y
Hubbard, M
Hubbe, M
Hubicki, C https://orcid.org/0000-0002-2092-3772
Hughes, C
Huh, Y
Humblot, V
Hunter-Adams, J
Hurley, L
Husen, A
Hussain, M
Hussein, SA
Hutchinson, C
Hutchinson, J
Hutson, K
Huyseune, A
Hwang, DS
Hyett, G
Ichiyanagi, K
Igl, L http://orcid.org/0000-0003-0530-7266
Ihara, Y
Ikegami, T
Ilanko, S
Ilany, A
Ilvonen, J http://orcid.org/0000-0002-2311-7039
Imran, M
Ino, K
Ioannou, C http://orcid.org/0000-0002-9739-889X
Ipek, O
Iranzo, J http://orcid.org/0000-0002-4538-7726
Ireland, P
Isella, A
Ishida, S
Islam, S
Ismadji, S
Jaatinen, K
Jablonka, E http://orcid.org/0000-0002-4549-8063
Jackson, B
Jackson, J http://orcid.org/0000-0002-4563-2840
Jackson, R
Jacobs, DS
Jafari, SM
Jain, G
Jain, H
Jalali, M
Jalili, M http://orcid.org/0000-0002-0517-9420
James, J
James, SC
Jandt, J http://orcid.org/0000-0002-0991-8577
Jani, A http://orcid.org/0000-0001-8719-2962
Janik, H
Janiszewska, M
Janvier, P
Jarvis, M
Jasra, A
Jayasinghe, S
Jeanniard du Dot, T http://orcid.org/0000-0003-1985-8325
Jeffery, K
Jenkins, A
Jenny, P
Jensen, P
Jernvall, J
Jiang, B
Jiang, F
Jiang, X
Jiménez-Muñoz, JC
Johanson, Z http://orcid.org/0000-0002-8444-6776
Johns, ED
Johnson, A
Johnson, C
Johnson, D
Johnson, E
Johnson, N
Johnston, R
Johri, A
Joice, R
Jolles, J http://orcid.org/0000-0001-9905-2633
Jones, A
Jones, G
Jones, M http://orcid.org/0000-0002-0146-9623
Jones, M
Jones, P https://orcid.org/0000-0001-7672-8397
Jordan, G http://orcid.org/0000-0002-6033-2766
Jorgensen, P
Joseph, J
Joumaa, V
Jovan, MN
Joyce, S
Ju, F
Jucker, C
Jung, D
Jung, J-L https://orcid.org/0000-0002-8795-8056
Jung, S http://orcid.org/0000-0002-1420-7921
Jungwirth, A
Jurado-Sanchez, B
Jurenka, R
Kaburu, S http://orcid.org/0000-0001-7456-3269
Kaczorowski, R
Kadilar, C
Kadmi, Y
Kahan, D
Kahn, R
Kaila, L
Kalantar-Zadeh, K
Kalckert, A
Kalevaru, N
Kalise, D
Kalnenieks, U
Kamerlin, SCL http://orcid.org/0000-0002-3190-1173
Kamya, P http://orcid.org/0000-0002-0135-9555
Kang, J
Kankala, R
Kao, A
Kara, HE
Karanjkar, P
Karásek, M http://orcid.org/0000-0002-8167-3009
Karl, S
Karlson, A http://orcid.org/0000-0001-6493-9533
Karnowski, K
Karpenko, I
Karsai, M
Kartha, KPR
Karthikeyan, S
Kassan, M
Katamssadan, T
Kauffman, M
Kausrud, KL
Kavaliers, M http://orcid.org/0000-0003-2292-5963
Kearns, C
Keesing, J
Keivanidis, P
Kekkonen, M http://orcid.org/0000-0001-7906-1698
Keller, R
Kennedy, D
Kenney, M
Kerbiriou, C http://orcid.org/0000-0001-6080-4762
Kessler, S
Kestler, H
Keupp, S http://orcid.org/0000-0002-5451-4256
Khalvati, A
Khamis, E
Khammash, M
Khan, LU
Khan, M
Khandelwal, M http://orcid.org/0000-0003-0368-3188
Khorsandi, S
Kiew, R
Kim, D
Kim, F
Kim, J-M
Kim, J
Kim, M
Kim, S-Y
Kim, Y
Kinoshita, M
Kinzer-Ursem, T
Kirby, D http://orcid.org/0000-0002-0469-413X
Kirsanov, D
Kirsch, I
Kirsch, L
Kissui, B
Kittakoop, P
Kiukas, J
Klapper, I
Kleidon, A
Klimek, P
Knörnschild, M
Knothe Tate, M
Knowles, P
Koeppl, C
Koerner, L
Komlos, J
Konečná, M
Konstantinidis, E
Koopman, J
Korn, A
Kornell, N
Korotaev, V
Korrensalo, A
Korving, L http://orcid.org/0000-0003-4753-0140
Koski, S
Koster, E
Koszytkowska-Stawińska, M
Koyama, N
Koyuncu, N
Krasheninnikova, A
Krasnov, A
Krasnov, K
Krawczyk, M
Krawczyk, T
Krcmar, S
Kristensen, AR http://orcid.org/0000-0002-5212-035X
Kristoufek, L
Krol, E
Krott, A
Krout, M
Krumhansl, C
Krupenye, C http://orcid.org/0000-0003-2029-1872
Ksepka, D
Kubinyi, M
Kuehn, C
Kuhlwilm, M http://orcid.org/0000-0002-0115-1797
Kumai, R
Kumar, M
Kumar, N
Kumar, R
Kumara, HN
Kundu, A
Kung, C-W
Kuntner, M
Kuroda, K
Kurvers, R
Kusumaatmaja, H
Kuzuya, A http://orcid.org/0000-0003-0003-2996
Kypraios, T
Labonte, D http://orcid.org/0000-0002-1952-8732
Lacarbonara, W http://orcid.org/0000-0002-8780-281X
Lacasa, L
Lachlan, R
Ladner, J
Lahey, B
Laine, E
Lakes-Harlan, R
Lakshmanan, VK
Lam, F-P
Lambert, M
Lambert, M http://orcid.org/0000-0003-3618-7260
Lambert, O http://orcid.org/0000-0003-0740-5791
Lambiotte, R
Lamboj, A
Lameris, T
Lammers, M http://orcid.org/0000-0002-7215-4426
Lan, S
Lan, W
Lander, M
Lang, A
Lange, M
Langton, S
Lany, N
Larkum, A
Larsen, O
Laruelle, S
Lashkari, N
Laskowski, K http://orcid.org/0000-0003-1523-9340
Late, D
Latty, T
Latzman, R
Laurin, M
Lavrinenko, A
Lawn, B
Leal, M
Leavens, D
Leavitt, A
Lécureuil, C
Lee, CF http://orcid.org/0000-0003-4363-9050
Lee, D-C
Lee, J
Lee, J
Lee, P
Lee, S http://orcid.org/0000-0001-6591-6464
Lee, YR
Legendre, P
Lehmann, G
Lehnertn, N
Lei, L
Leite-Almeida, H
Leman, M
Lenardon, M
Lenormand, M
Leopold, D
Lessells, R
Lesyk, R
Leung, C
Levchenko, A
Levin, M http://orcid.org/0000-0001-7292-8084
Lewis-Smith, H
Lewsey, M
Ley, K
Leyser, O
Li, P http://orcid.org/0000-0001-8771-3369
Li, B
Li, B
Li, F
Li, G
Li, K
Li, N
Li, P
Li, Q
Li, S
Li, S
Li, W
Li, X
Li, X https://orcid.org/0000-0002-4115-3287
Li, X
Li, X
Li, Y
Li, Y
Li, Y
Li, Y
Li, Z
Li, Z
Liang, H
Liang, W
Liang, Z
Liarokapis, M
Lichtwark, G
Liebgold, E
Liegeois, F https://orcid.org/0000-0002-8640-6217
Liljenström, H
Lilley, M
Lillig, CH
Lilllard, A
Lillywhite, H http://orcid.org/0000-0002-6410-4832
Lima, C
Lima, L
Lima, M https://orcid.org/0000-0001-5540-8802
Lima, S
Lin, B
Lin, F
Lin, W
Lin, Y
Linclau, B
Lindahl-Jacobsen, R
Lindgren, BS
Linding, R
Lindner, E
Lindroos, M
Lindsay, S https://orcid.org/0000-0002-6439-987X
Linko, V
Lion, S http://orcid.org/0000-0002-4081-0038
Litton, C
Liu, A
Liu, B
Liu, H
Liu, H
Liu, J
Liu, J
Liu, J
Liu, J
Liu, J
Liu, P
Liu, Q
Liu, W http://orcid.org/0000-0001-6472-6247
Liu, X
Liu, X
Liu, X
Liu, X
Liu, X
Liu, X
Liu, YJ
Liu, Y
Liu, Y https://orcid.org/0000-0003-2211-3535
Liu, Y
Liu, Z
Liu, Z
Liu, Z-H
Liyanage, T
Lloyd, G http://orcid.org/0000-0001-6887-3981
Lobaskin, V
Lobel, A
Lockhart, T
Lodziana, Z
Loh, XJ http://orcid.org/0000-0001-8118-6502
Loiseau, C
Lombard, B http://orcid.org/0000-0002-5648-2737
Longmore, C
Longo, A
Lonzarich, G
Lopez, C
Lopez Gonzalez, C
Lopez, M
López, M
López-Arbarello, A http://orcid.org/0000-0002-2924-3319
Lopez-Garcia, P http://orcid.org/0000-0002-0927-0651
Lopman, B
Lord, C
Lord, K
Lorenzo, R
Loveridge, N
Lowe, A
Lu, Y
Luboobi, L
Lucan, R
Lucas, E
Lucas, M
Lucena, R
Lucon-Xiccato, T
Ludeke, S
Luders, U
Luebert, F
Luis, S
Lukas, D http://orcid.org/0000-0002-7141-3545
Lukashkin, A
Luo, H
Luo, S
Lusher, A
Lv, X
Lvov, Y
Lynch, J
Lyons, S
Ma, A
Ma, G
Ma, J
Ma, R
Ma, W
Ma, Y
Ma, Z
Macdonald, T
Mackay, R
Maclaren, I
MacLean, E
MacNulty, D
Macy, M
Madej, K
Maden, M
Madhusudan, P
Madzvamuse, A http://orcid.org/0000-0002-9511-8903
Magnotti, J http://orcid.org/0000-0003-2093-0603
Mahmood, K
Mahmudov, K
Maia, A
Majid, A http://orcid.org/0000-0003-0132-216X
Makbol, N
Malcolm, P
Maldonado, Jhttps://orcid.org/0000-0002-4282-1072
Malik, A
Malucelli, G
Mamedov, B http://orcid.org/0000-0002-8398-9170
Manaka, T
Manassa, R
Manchein, C
Mandalaywala, T
Mandolini, L
Mann, S
Manser, M
Manuel Zamaro, J
Manzello, D
Mao, Z
Mao, C
Marcus, Y http://orcid.org/0000-0002-3138-9704
Mareschal, D
Margetic, D
Mari, J-M
Marín, S http://orcid.org/0000-0002-0714-6155
Marjoram, P
Marples, N
Marquez Prieto, J
Marsh, B
Marsh, L
Marshall, D
Marshall, J
Martén-Rodríguez, S
Martin, A
Martin, L
Martin, S
Martinez, L
Martinez-de la Cruz, A
Martius, C https://orcid.org/0000-0002-6884-0298
Marucci, L
Marugán-Lobón, J
Marusyk, A
Mascolo, C
Mash, D
Mashima, K
Maslivets, A
Mason, P http://orcid.org/0000-0002-5143-666X
Maspoch, S
Masuda, N http://orcid.org/0000-0003-1567-801X
Mather, W
Mathger, L http://orcid.org/0000-0002-0603-0345
Mathis, A
Mathur, R
Matras-Postolek, K
Matson, C
Matsuo, Y
Matthews, B
Matthews, L
Mattison, S
Matzke, N
Mawson, A
Maxwell, S
May-Collado, L
Mayr, G http://orcid.org/0000-0001-9808-748X
Mbaiwa, F
Mboera, L
McAuliffe, K http://orcid.org/0000-0002-4230-0250
McCall, M
McCallum, H
McCarthy, R
McClain, C http://orcid.org/0000-0003-0574-428X
McClintock, J
McCluskey, A http://orcid.org/0000-0001-7125-863X
McCowan, B
McCue, M
McCullen, N
McDaniel, J
McElligott, A
McEwen, A
McGrady, J
McGruder, C
McHenry, M http://orcid.org/0000-0001-5834-674X
McHorse, B http://orcid.org/0000-0003-3715-812X
McKechnie, A
McKenzie, A
McKenzie, D
McMahon, R
McMullan, M
McQuillan, I
Meade, T
Medina, J
Meemken, F
Meenakshi, M
Megill, W
Meguid, S http://orcid.org/0000-0001-7151-9932
Mei, W
Meijer, H
Meijles, D http://orcid.org/0000-0002-5557-3549
Meirelles, LA http://orcid.org/0000-0003-3194-7136
Meissner, M
Mejuto, J
Melaibari, A
Melde, B
Meldon, J
Memboeuf, A
Memmott, J
Méndez-Sánchez, R
Meng, T http://orcid.org/0000-0002-6507-7661
Menzel, CR
Mergeay, J
Metcalfe, C
Metcalfe, N
Metzakopian, E
Meule, A
Meyer-Rochow, V
Mhaske, SB
Michalek, A
Micheli, D
Middleton, S
Mielke, A
Miko, I
Millard, T
Miller, C
Miller, G
Miller, M
Mills, K
Milne, A
Miner, B
Minetti, A
Ming, J
Minghetti, M
Minton, R
Mirams, G
Mishra, S
Missoni, S
Mitchell, J
Mitoraj, MP
Mitra, S
Mitre, E
Mitrovic Dankulov, M
Mitrovic, M
Mittal, S http://orcid.org/0000-0003-0799-328X
Mitton, J
Miyata, S
Miyatake, T
Mjolsness, ED
Mobin, S
Modesto-Lopez, LB
Moffett, B
Moggach, S
Mohammad, A http://orcid.org/0000-0002-0691-3199
Mohana Roopan, S
Mokkapati, S
Molla, M
Møller, A http://orcid.org/0000-0003-3739-4675
Moller, M
Molnar, J
Mondragon, R
Monechi, B http://orcid.org/0000-0003-3841-3989
Moodley, B
Mooers, A
Moore, A http://orcid.org/0000-0002-1498-3322
Moore, M
Mooring, M
Moors, A
Moraes, C
Morfu, S
Morgan, E
Morgenroth, T
Morgenstern, B
Moritz, T
Morresi, T
Morrill, L
Morriss, J
Moseby, K
Moses, E
Motta, P
Mouchet, Shttp://orcid.org/0000-0001-6611-3794
Mougi, A http://orcid.org/0000-0002-0902-758X
Moura, A
Mouret, JB
Moyà-Solà, S
Mubayi, A
Mueller, S
Muggeridge, A
Mughal, S http://orcid.org/0000-0002-1675-489X
Mukherjee, B
Mukherjee, S
Mukherjee, S
Mulholland, A
Muller, U
Mulville, J
Munclinger, P
Mundaca, E
Munday, P
Munga, S
Murayama, K
Murch, G
Musilova, Z
Musolesi, M
Mustoe, A
Mutalik, S
Muthamilarasan, M
Muthukrishna, M
Mychajliw, A
NA, A
Naftaly, M
Nager, R
Naik, R
Naikade, NK
Nakabachi, A
Nakajima, T
Nakamaru, M http://orcid.org/0000-0003-2116-1711
Nakamura, N
Nakanishi, Y
Nakata, K http://orcid.org/0000-0002-1207-3448
Nakatsukasa, M
Nakazawa, Y
Nalam, P
Nalin De Silva, KM http://orcid.org/0000-0003-3219-3233
Nallim, L
Nanda, S
Nandi, P
Nardell, E
Narracott, A
Narula, J
Naskar, AK
Natalini, R
Nathke, I
Nawroth, C http://orcid.org/0000-0003-4582-4057
Nazare, M
Neda, Z
Nelson, C
Nesbitt, W
Nesje, A
Neu, J
Neumann, C http://orcid.org/0000-0002-0236-1219
Neumeyer, C
Neves, I
Neville, A
Newcomb, J
Newheiser, A-K http://orcid.org/0000-0001-7006-2096
Nico, L
Niculescu, V-C
Nidheesh, PV
Nielsen, A http://orcid.org/0000-0001-6554-8358
Niemuth, N
Nizovtseva, I http://orcid.org/0000-0002-7766-7351
Noble, L
Noda, R
Noda, T
Noda, Y
Noonan, S
Nordon, R
Noren, D http://orcid.org/0000-0002-9544-4530
Norris, R
Novas, F
Novelli, M
Novozhilov, A
Nowak, M
Nowell, D
Nowicki, S
Nummela, S
Nurunnabi, M
Nussear, K
Nuttall, W
Nyhan, B http://orcid.org/0000-0001-7497-1799
Oberst, J
Obot, IB
O'Brien, M
O'Connor, T
Ogawa, S
Oghalai, J
Ogneva-Himmelberger, Y
Ogunlade, O
Ohtsuka, S
Oigiangbe, N
Okamura, H
Okasha, S
Okuyama, J
Oladoja, N
Olah, K
Oliveri, V
Olson, S
Omachi, H
O'Mara, T
Omoruyi, F
O'Mullane, A
Ong, W-J http://orcid.org/0000-0002-5124-1934
Opell, B http://orcid.org/0000-0002-1830-0752
Ordano, M http://orcid.org/0000-0003-0962-973X
Ornelas, JF
Ortega-Jiménez, V http://orcid.org/0000-0003-0024-5086
Orton, D
Ostrom, P http://orcid.org/0000-0002-9953-8809
Otanicar, T
Otero, A
Ouldridge, T
Outram, A
Overcast, I
Øverli, Ø
Owais, M
Owens, G
Oyen, M http://orcid.org/0000-0002-3428-748X
Oyetunji, O
Ozel, G
Oziolor, E
Özkir, D
Ozturk, HK
Padfield, D
Padilla-Gamino, J http://orcid.org/0000-0003-0478-9953
Padula, V
Page, M
Paiva, V
Pal, A
Pal, S
Pallares, L
Palmer, C http://orcid.org/0000-0002-1058-3428
Pan, H
Pan, Y
Pancerz, K
Pandey, G
Pang, J http://orcid.org/0000-0001-6965-4166
Pankratz, M
Pantazis, P
Pantelis, N
Papadakis, R http://orcid.org/0000-0001-9734-7550
Papastamatiou, Y http://orcid.org/0000-0002-6091-6841
Paquay, S
Pardo, L
Parikh, M
Parisod, C
Parkesh, R
Parkinson, J
Parkkinen, J
Parks, S
Parmigiani, S
Parncutt, R
Parra, M
Paryani, R
Passow, C
Pastore, H
Patel, J https://orcid.org/0000-0002-2357-7596
Patel, S
Patra, D
Patrocinio, AOT
Paul, C
Paul, N
Paula, J
Paule, J
Pawlowski, B
Payne, A
Pearce, P http://orcid.org/0000-0001-5788-3826
Peel, A
Peila, D
Pellis, L
Peng, T
Penhune, V
Pennel, T
Penta, R
Pepin, K http://orcid.org/0000-0002-9931-8312
Perc, M http://orcid.org/0000-0002-3087-541X
Percival, D
Pereira, L
Peremans, H
Pérez Izquierdo, A
Pérez Mellado, V
Pereza, N
Pérez-Matus, A
Perez-Pariente, J
Pérez-Rigueiro, J
Perez-Rodriguez, L http://orcid.org/0000-0002-5926-1438
Perez-Tris, J
Perez-Velazquez, J
Performs, A
Perillo, M
Perkoulidis, G
Perra, N
Perret, M
Perrett, D
Petermann, I
Peters, M
Peters, S
Petersen, A http://orcid.org/0000-0002-0955-3483
Petit, O
Pfattheicher, S
Pfennig, K http://orcid.org/0000-0002-0852-287X
Pfenninger, M
Phan, R
Phillips, A
Pike, A
Pike, T http://orcid.org/0000-0002-6942-0498
Pilakouta, N
Pilastro, A http://orcid.org/0000-0001-9803-6308
Pilgrim, E
Pillai, S
Pillay, V
Pin, C
Pinheiro, D
Pintor, L
Pizlo, Z
Planat, M http://orcid.org/0000-0001-5739-546X
Plank, M
Plath, M
Platt, M
Plotnik, J http://orcid.org/0000-0002-7597-8818
Plunian, F
Podlewska, S
Polly, P
Pomara, L
Pommerening, A
Pongracz, P http://orcid.org/0000-0001-5126-299X
Ponton, F
Poon, W
Porfiri, M http://orcid.org/0000-0002-1480-3539
Portegies Zwart, S
Porter, M
Potier, P http://orcid.org/0000-0003-3156-7846
Powers, D http://orcid.org/0000-0003-1126-7141
Prabakar, K
Prada-Gracia, D
Prato-Previde, EP
Pratt, S
Pratviel, G
Pregnolato, M
Preis, T
Price, D
Price, D
Price, E http://orcid.org/0000-0001-6042-7020
Pringle, C
Pritchard, V
Pritsch, K
Protopopova, A
Proulx, M
Przybylski, A http://orcid.org/0000-0001-5547-2185
Pulkkinen, K
Pumera, M
Punyasena, S
Puranik, A
Purkait, T
Putman, N http://orcid.org/0000-0001-8485-7455
Qi, R
Qian, L
Qiao, W
Qiu, J
Quistberg, A
Quraishi, MA
Qureshi, N
Saravanan R http://orcid.org/0000-0002-3771-4694
Rabalais, N
Rabi, M
Radhakrishnan, S
Radi, E
Raesaenen, K
Rafatullah, M
Rafiq, MA
Rafter, M
Ragauskas, A
Rage, J-C
Raghunathan, T
Raguso, R
Rahel, F
Rahnev, D
Rajpurohit, S
Ramadan, M
Ramaswamy, K
Ramirez, J
Ramkrishna, D
Rana, D
Ranjith, P http://orcid.org/0000-0003-0094-7141
Rao, C
Rao, S
Raposo, E
Rasapalli, S
Rashidi, H http://orcid.org/0000-0001-8078-6688
Rauthmann, J
Raval, M
Raverty, S
Ray, J http://orcid.org/0000-0001-7968-0851
Ray, S
Raza, H
Read, A
Reardon, E
Rebula, J
Reckin, R
Reddy, MS
Reid, M
Reina, A http://orcid.org/0000-0003-4745-992X
Ren, H
Ren, Y
Rendell, L http://orcid.org/0000-0002-1121-9142
Renesto, S
Rensel, M
Repo, T
Resing, J
Retterer, S
Reynaud, S
Reynolds, A http://orcid.org/0000-0002-7103-3841
Reynolds, Q
Rezlescu, C
RIBE, N
Ribeiro, HV http://orcid.org/0000-0002-9532-5195
Ribeiro, J
Rice, K
Richard Webber, R
Richards, P
Richardson, J
Riche, M
Rideout, E
Ridley, A
Riebel, K
Riffo, V
Rigato, S
Rinaldo, A
Ritzoulis, C
Robertson, B
Robertson, M
Robinson, G
Roblitz, S https://orcid.org/0000-0002-2735-0030
Rocha, L
Rodgers, E
Rodgers, R
Rodgers, T
Rodrigues, A
Rodrigues, L
Rodriguez, N
Rodriguez, R
Rodriguez, S
Roelofs, D
Rogell, B http://orcid.org/0000-0002-5553-2691
Rogers, A
Rohland, N
Rohlfs, M
Rojas, B http://orcid.org/0000-0002-6715-7294
Rokhum, L http://orcid.org/0000-0001-8820-569X
Roles, A
Rollins-Smith, L
Romanelli, L
Romea, P
Romey, W http://orcid.org/0000-0003-2759-6791
Rong, Z
Rosas, F
Rosel, P
Rosenthal, M http://orcid.org/0000-0003-1291-1728
Rosenzweig, M
Rossetto, M
Rossion, B
Roth, S
Rotics, S
Rotllant, G
Rousset, F
Rowe, L
Rowland, H http://orcid.org/0000-0002-1040-555X
Ruan, J
Rubí, M
Rubin, K
Rudy, K
Ruell, E
Rufat, S
Ruiz, J
Ruiz, M
Ruiz-Lara, S
Rundel, P
Rusch, H http://orcid.org/0000-0002-9260-4025
Russo, D
Russo, M
Rydell, J http://orcid.org/0000-0002-4930-6110
Ryu, J-H
Saarela, O
Sacco, I
Sachdev, A
Saffran, J
Saini, A
Sakai, K
Saleem, A
Saltzman, W
Salvanes, AGV
Sanchez-Duarte, R
Sandin, S
Sansom, R http://orcid.org/0000-0003-1926-2556
Santhanakrishnan, A
Santiesteban, I
Sapir, N
Saremi, M
Sari, E
Saroglou, V
Sasaki, Y
Sato, T
Satta, Y
Saupe, E
Sava, G
Savion, N
Sayigh, L http://orcid.org/0000-0001-8334-1326
Sayyaadi, H
Scampicchio, M
Scanlon, D
Scarpino, S
Scarponi, D
Schakner, Z http://orcid.org/0000-0002-8325-3526
Schausberger, P http://orcid.org/0000-0002-1529-3198
Scheel, A http://orcid.org/0000-0002-6627-0746
Scherrmann, M-C
Scheyd, G
Schindler, K
Schindler, S
Schlinger, B
Schlöder, J
Schloegl, C
Schlötterer, C
Schmidt, B http://orcid.org/0000-0002-4023-1001
Schmidt, W
Schmitt, M
Schneider, HG
Schock, T
Schoefs, B http://orcid.org/0000-0002-2468-0659
Schoepf, V http://orcid.org/0000-0002-9467-1088
Schofield, A
Schofield, G
Scholtyssek, C
Schraagen, JM
Schreiber, E
Schroeder, M
Schulte-Rüther, M
Schultz, Z
Schultze, HP
Schulz, A
Schulze, K
Schum, A
Schuster, S http://orcid.org/0000-0003-2828-9355
Schuwerk, T http://orcid.org/0000-0003-3720-7120
Schwartz, I
Schwartz, T http://orcid.org/0000-0002-7712-2810
Schwimmer, D
Scott-Samuel, N
Scully, AD
Seal, A
Searle, J
Searle, K http://orcid.org/0000-0003-1316-9342
Secomb, T
Seenivasan, R
Seethamraju, S
Segawa, E
Seibel, B
Seidl, A
Seker, UOS
Self, Z http://orcid.org/0000-0003-1419-8735
Senetakis, K http://orcid.org/0000-0003-0190-4768
Sereno, P
Servia-Rodriguez, S
Seyfarth, R
Seymour, M
Seymour, R http://orcid.org/0000-0002-3395-0059
Shahar, R http://orcid.org/0000-0002-3684-1800
Shandarin, S
Shanks, D
Shannon, H
Shao, Q
Shao, Z
Sharma, S
Sharma, V
Sharon, G
Shashar, N
Shehzad, K
Sheka, D
Shelton, D
Shepard, E
Sheppard, P
Shepperson, B
Sherley, R http://orcid.org/0000-0001-7367-9315
Sherratt, E http://orcid.org/0000-0003-2164-7877
Sherratt, T http://orcid.org/0000-0001-6837-3848
Shi, G
Shi, X
Shi, Y
Shiffrin, R
Shillcock, R
Shimpi, M
Shirinifard, A
Shreeve, T
Shuker, D
Shultz, S http://orcid.org/0000-0002-7135-4880
Si, K
Si, Y
Siddiqui, MRH
Sieck, S
Siemens, J
Sievert, S
Signor, S http://orcid.org/0000-0003-2401-0644
Signorella, S
Silitonga, AS
Silk, M http://orcid.org/0000-0002-8318-5383
Simini, F http://orcid.org/0000-0001-8675-3529
Simmons, B
Sinatra, R
Sindbæk, S
Singh, A http://orcid.org/0000-0002-1451-2838
Singh, B
Singh, P
Singh, P
Singh, S
Singh, S
Singhal, N
Siniscalchi, M http://orcid.org/0000-0002-1592-6549
Sinniah, K
Situ, H
Sivak, D
Sknepnek, R
Skoglund, P
Sladkowski, J
Slager, R-J
Slaughter, V http://orcid.org/0000-0001-9315-1497
Slijepcevic, P
Smaldino, P http://orcid.org/0000-0002-7133-5620
Small, M http://orcid.org/0000-0001-5378-1582
Smith, C
Smith, D
Smith, G
Smith, L
Smith, M http://orcid.org/0000-0001-5660-1727
Smithson, T http://orcid.org/0000-0002-6546-1145
Smolla, M http://orcid.org/0000-0001-6367-8765
Snell, T https://orcid.org/0000-0001-6175-1535
Snijders, L
Snively, E
Soares de Andrade, J
Soares, B
Soberon, M
Sobhi, M
Sobkowicz, P
Soderstrom, M
Sogard, S
Soibelzon, L
Soininen, J
Solomon, A
Solomon, J
Somers, A
Somerville, A
Sommer, W
Song, J http://orcid.org/0000-0003-2821-9429
Song, S http://orcid.org/0000-0002-5318-6705
Song, Y http://orcid.org/0000-0002-0760-8591
Song, Y
Soriano-Fradera, J
Sosa, S
South, M
Sparkes, T
Spellman, B
Spence, A
Spitzer, B
Spotila, J
Sramko, G
Sreeram, KJ
Sridhar, A
Srinivasarao, M
Srivastava, N
Srivatsan, S
Srygley, R
St. Hilaire, M
Stanish, C
Stayton, T
Steadman, D
Steedman, M
Steele, J
Stefanova, V
Steiger, S
Stein, L
Steinbauer, MJ
Steinman, S
Steinmetz, R
Steinruck, H
Stephan, C
Sterelny, K http://orcid.org/0000-0003-3159-6698
Sterli, J
Sternberg, E
Stevenson, P
Stewart, I
Stimpson, C
Stockle, M
Storrs, GW
Strader, M
Strassmann, J
Straub, T
Strausfeld, N
Strauss, W
Strogatz, SH
Ströhl, F
Stroustrup, N
Stuber, E
Sturdy, C
Stutchbury, B
Styles, P
Su, P
Su, L
Su, L
Su, Q
Subalusky, A
Subramanian, B
Suginome, M
Sugiyama, S
Suizu, K
Sullivan, N
Sullivan, R
Sun, G
Sun, L
Sun, X
Sun, Z
Sundstrom, A http://orcid.org/0000-0002-3378-4513
Surbeck, M http://orcid.org/0000-0003-2910-2927
Sutton, G
Suvarnaraksha, A
Suzuki, M
Suzuki, M
Swanson, B
Swart, A
Swei, A
Swiderski, D
Szabo, A
Szabo, G
Sze Teng, L
Szeto, KY
Szolnoki, A
Szulkin, M
Taboada Sotomayor, MdelP
Taglietti, A http://orcid.org/0000-0003-0141-9201
Takeshita, F
Takeuchi, T http://orcid.org/0000-0002-5641-2333
Talhelm, T https://orcid.org/0000-0002-0954-5758
Talyzin, AV
Tammaru, T
Tan, F
Tan, M
Tan, X
Tan, X
Tanaka, M http://orcid.org/0000-0003-2198-1402
Tanaka, M
Tang, W
Tang, X
Taniguchi, I
Tanskanen, A
Tanvir, N
Tao, L
Tao, T
Tapia, G
Tapia-Paniagua, S
Tate, AT http://orcid.org/0000-0001-6601-0234
Taubert, J http://orcid.org/0000-0002-6519-8068
Taugbøl, A http://orcid.org/0000-0003-1295-5675
Tavčar, G
Taylor, A
Taylor, A http://orcid.org/0000-0003-0071-8306
Taylor, D
Taylor, J http://orcid.org/0000-0001-6461-0869
Taylor, J https://orcid.org/0000-0002-1109-8539
Tecilla, P
Tegeder, P
TeGrotenhuis, W
Tehrani, J
Teichroeb, J
Tejero, J
Tekir, S
Tellez, G
Tennant, D
Teodoriu, HC
Tessone, C
Thakar, J
Thakar, J
Theraulaz, G http://orcid.org/0000-0002-3062-4215
Thewes, M
Thickett, SC
Thielges, M
Thierry, B
Thiv, M
Thode, A
Thom, M https://orcid.org/0000-0002-9985-0157
Thomas, J
Thomas, S
Thomas, SP
Thompson, S
Thorgaard, G
Thornton, A
Thorogood, R
Thouvarecq, R
Thrush, S http://orcid.org/0000-0002-4005-3882
Thuenken, T
Thuy, B
Tian, X (Cindy)
Tibshirani, R
Tighe, C
Tobias, J http://orcid.org/0000-0003-2429-6179
Todrank, J
Tofanica, B
Toigo, A
Toivianen, P
Tojo, C
Tolimieri, N
Tommaso, T
Tomuta, I
Toro, J
Torres-Florez, JP
Toth, M
Toyokawa, W http://orcid.org/0000-0001-8558-8568
Tragust, S
Tramier, M
Tranmer, M
Tremblay, L
Tremblay, Y
Tripathi, B
Troscianko, J
Trzeciak, A
Tschinkel, W
Tsuji, T
Tu, M
Tudorache, C
Tulkki, J
Tummino, ML
Tupper, P
Turco, E http://orcid.org/0000-0002-8263-7034
Turin, L
Turner, J
Turner, N
Tuzimski, T
Tyne, J http://orcid.org/0000-0002-0676-5659
Typas, M
Tzakos, AG
Tzou, Y-M http://orcid.org/0000-0002-3680-7303
Tzourio-Mazoyer, N
Ueda, M
Uggla, C http://orcid.org/0000-0003-1639-3307
Uhen, M
Uher, J http://orcid.org/0000-0003-2450-4943
Umar, A
Unckless, R
Unsworth, W
Upchurch, P
Urazghildiiev, I
Uroos, M
Usherwood, J http://orcid.org/0000-0001-8794-4677
V. Pergal, M
Vácha, R https://orcid.org/0000-0001-7610-658X
Vaezi, M
Vaiman, D http://orcid.org/0000-0002-1915-0717
Vale, G
Valente, J
Valiyaveettil, S
Valkonen, J
Van der Meer, W
Van Doninck, K
Van Dyke, J
van Elk, M
Van Heugten, M
Van Schaik, J http://orcid.org/0000-0003-4825-7676
Van Vuren, D
Vanderelst, D
Vanhove, M
Vanwanseele, B
Varenberg, M http://orcid.org/0000-0001-5552-0511
Vargas-Bernal, R
Vashist, S
Vasilescu, A
Vaudo, J
Vedrine, J
Vega, N
Vega, V
Vehrencamp, S
Veling, H
Velotta, J
Venkadesan, M http://orcid.org/0000-0001-5754-7478
Vera, M
Verboord, M
Vereecke, E
Vergani, L http://orcid.org/0000-0003-2353-7751
Verhulst, E
Verkoeijen, P
Verma, JP
Vermeij, G http://orcid.org/0000-0002-6450-5687
Veselý, P
Vial, C
Victor, M
Vieira, E
Vigilant, L
Vihman, M
Vijayakumar, S http://orcid.org/0000-0002-4210-2959
Vilà, C
Vilar, M
Vincent, B
Vincent, M
Vines, P
Viney, M http://orcid.org/0000-0002-4857-1238
Viswanathan, G
Vladimir, N
Voets, T
Vogel, D
Vogel, KJ
Voliotis, M
Vøllestad, L
Voloshina, A
Vorobyev, M http://orcid.org/0000-0001-7615-5816
Voss, H
Voss, K
Voyiadjis, G http://orcid.org/0000-0002-7965-6592
Vroomen, J
Vulpe, C
Vyvyan, J
Wagoner Johnson, A
Wakano, J
Wake, G
Waldenström, J
Waldman, B http://orcid.org/0000-0003-0006-5333
Walker, S
Wallace, C
Wallace, V
Waller, D
Walsh, A
Walter, J http://orcid.org/0000-0003-2983-751X
Waltert, M
Wang, H
Wang, C-T
Wang, C
Wang, C-J
Wang, D
Wang, F
Wang, F
Wang, F
Wang, G-L
Wang, H
Wang, H http://orcid.org/0000-0003-1170-0160
Wang, J
Wang, J https://orcid.org/0000-0001-7037-9368
Wang, K
Wang, M
Wang, Q
Wang, Q
Wang, R-T
Wang, S
Wang, S
Wang, S
Wang, S
Wang, S-J
Wang, T
Wang, W-X
Wang, X
Wang, X
Wang, X
Wang, X
Wang, X
Wang, Y http://orcid.org/0000-0002-7576-0907
Wang, Y
Wang, Z
Wang, Z
Wang, Z
Wang, Z
Wang, Z http://orcid.org/0000-0003-1669-4548
Wang, Z
Wang, Z
Waples, R
Wappler, T
Ward, A http://orcid.org/0000-0003-0842-533X
Ward, J
Ward-Thompson, D
Warnow, T
Warrick, D
Warwick, MEA
Wasmuth, J
Wasser, S
Waters, J http://orcid.org/0000-0001-7077-9441
Waters, J http://orcid.org/0000-0002-1514-7916
Weatherley, L
Webb, J http://orcid.org/0000-0003-4822-6829
Weber, M
Webster, T
Wee, LH
Wei, G
Wei, J-R
Wei, J
Wei, Z-J http://orcid.org/0000-0003-1729-209X
Weinstock, J
Weisbecker, V
Weiss, A http://orcid.org/0000-0002-9125-1555
Weiss, R
Weiss, S
Welcomme, R
Wen, H
Wendrich, W
Werner, B
Wertheimer, A
Westenberg, M
Westneat, DF
Westneat, M
Wetzel, D
Wey, T
Wharton, J
Whitaker, D http://orcid.org/0000-0003-0674-4417
White, D http://orcid.org/0000-0002-6366-2699
White, F
White, T http://orcid.org/0000-0002-3976-1734
Whiting, S
Wiedemann, L
Wiegrebe, L http://orcid.org/0000-0002-9289-6187
Wiertlewski, M
Wikenros, C https://orcid.org/0000-0002-2825-8834
Wilby, P
Wilkerson, R
Wilkinson, K
Williams, A
Williams, C
Williams, M
Willis, K
Willner, I
Willocquet, L
Willwacher, S http://orcid.org/0000-0002-1303-3165
Wilson, A
Wilson, D
Wilson, L
Wilson, R
Wilt, J
Wilts, B http://orcid.org/0000-0002-2727-7128
Winberg, S
Windmill, J http://orcid.org/0000-0003-4878-349X
Winfield, A
Winnefeld, F
Winters, A http://orcid.org/0000-0003-2840-6267
Wissem, M
Withayachumnankul, W
Withers, P
Witte, K
Wittig, R
Wittmer, J
Wohl, C
Wohlrabe, K
Wolf, C
Wolf, S http://orcid.org/0000-0002-3747-8097
Wolf, V
Wolfram, M-T http://orcid.org/0000-0003-1133-8253
Wolgemuth, CW
Wong, J
Wong, M
Wong-Ng, W
Woolfson, DN http://orcid.org/0000-0002-0394-3202
Wooten-Eagle, J
Work, T
Wrangham, R
Wright, G
Wright, G https://orcid.org/0000-0001-9424-5743
Wu, G
Wu, G
Wu, H
Wu, JH
Wu, J
Wu, L
Wu, S
Wyatt, A
Wyneken, J
Wynn, K
Wynne, C
Wyse, J
Xia, BY
Xia, J
Xia, X
Xia, Y-J
Xiang, S
Xiao, F
Xiao, G
Xiao, T
Xie, J
Xie, L
Xin, L http://orcid.org/0000-0002-4683-4612
Xiong, H
Xiong, J
Xu, B
Xu, C
Xu, C
Xu, L-W
Xu, L
Xu, N
Xu, X
Xu, X
Xu, X-W
Xu, Y
Xu, Z
Xu, Z
Xue, F
Yackulic, C
Yahui, H
Yamada, Y
Yamamoto, K
Yamanashi, Y
Yamasaki, H
Yamashita, A
Yamazaki, S
Yamazaki, Y
Yan, J-K
Yan, X
Yang, B
Yang, C
Yang, C
Yang, C-Y
Yang, C
Yang, J
Yang, J
Yang, L
Yang, P
Yang, S
Yang, W
Yang, X
Yang, Y
Yang, Z
Yang, Z
Yao, C
Yao, G
Yao, H http://orcid.org/0000-0003-0549-2246
Yao, P
Yao, Z
Yashar, A
Yasseri, T http://orcid.org/0000-0002-1800-6094
Yavuz, M http://orcid.org/0000-0003-3452-8551
Yi, J
Yıldız, T
Yilmaz, V
Yin, RH
Yin, X
Ying, Y
Yi-Tao, L
Yoon, H
Yoshida, Y
Yosipovitch, G
Young, S-J http://orcid.org/0000-0003-3164-2949
Yousefi, N
Yu, F
Yu, Q
Yu, W
Yuan, J-W
Yuan, M
Yuan, W
Yuan, Y
Yuan, Z
Yuasa, M
Yuca, E
Yun, S
Zachos, F
Zagoskin, A
Zalucki, M http://orcid.org/0000-0001-9603-7577
Zanin, M
Zanker, J
Zanusso, O
Zaretskiy, L
Zeder, M http://orcid.org/0000-0001-9716-4877
Zeh, J
Zeil, J
Zeng, L
Zettler, E
Zeugolis, D
Zhai, L-J
Zhai, S
Zhai, X
Zhang, C-Y
Zhang, D
Zhang, D
Zhang, D
Zhang, F
Zhang, F
Zhang, J
Zhang, J
Zhang, J
Zhang, J
Zhang, K
Zhang, L
Zhang, M
Zhang, P
Zhang, S
Zhang, X
Zhang, X
Zhang, Y
Zhang, Y
Zhang, Y
Zhang, Y
Zhang, Z
Zhao, J
Zhao, S
Zhao, S
Zhao, X
Zhao, Y
Zhao, Y
Zheng, A
Zheng, Q
Zheng, S
Zheng, Y
Zheng, Y
Zheng, Y
Zhi, C
Zhou, G
Zhou, L
Zhou, W
Zhou, X
Zhou, X
Zhu, D
Zhu, L
Zhu, L
Zhu, L
Zhu, W
Zhu, X
Ziegler, A
Zietsch, B
Zimmerman, M
Zink, R http://orcid.org/0000-0001-9243-4567
Zografopoulos, DC
Zou, X
Zuffanelli, S
Zuo, R
Zych, E
